# IMPACT OF SEX AND ANTIHYPERTENSIVE MEDICATION ON GLOBAL COGNITION IN PRIMARY CARE OLDER ADULTS

**DOI:** 10.1093/geroni/igac059.2533

**Published:** 2022-12-20

**Authors:** Adrian Noriega de la Colina, Helen-Maria Vasiliadis, Djamal Berbiche, Louis Bherer, Helene Girouard, Navin Kaushal

**Affiliations:** McGill University, Westmount, Quebec, Canada; Sherbrooke University, Longueuil, Quebec, Canada; Sherbrooke, Sherbrooke, Quebec, Canada; University of Montreal, Montreal, Quebec, Canada; University of Montreal, Montreal, Quebec, Canada; Indiana University, Indianapolis, Indiana, United States

## Abstract

Hypertension is one of the strongest modifiable risk factors for the development of cognitive impairment and dementia. However, there are conflicting reports regarding which class of antihypertensive medication is the best for reducing the risk of cognitive decline. The objective of this study is to determine whether sex determines the pharmacological therapy that is the most effective in preserving cognitive outcomes. This study examined 1607 participants from the ESA Services Study, a longitudinal survey of older adults over 65 years old in Quebec-Canada. They were examined for the Mini-Mental State Examination(MMSE) at baseline (T1) and followed up three (T2) and four years after (T3). Hypertensive women had the highest mean MMSE score at each time point (T1 28.591 (SE .064); T2 28.282 (SE .118); T3 28.524 (SE.119)), while hypertensive men had the worst (T1 28.038(SE.070); T2 27.694(SE.125); 27.809(SE.128)). Women taking angiotensin II receptor antagonists (ARBs) showed the highest MMSE scores (p<.003), and men taking diuretics and other antihypertensives had the lowest MMSE scores(p<.001) after a 3-year follow-up. Combination therapy of two or three antihypertensives drugs was associated with higher scores in women at T1 and T2 (p<.001). In men, taking three antihypertensives showed a sharp decrease in MMSE scores from T1 to T3 (p<.001). Sex differences in global cognition outcomes in older adults are in part due to the heterogeneity in effects related to the type and number of antihypertensive drugs used. Effective antihypertensive treatment should consider the impact of sex to optimize the effect of pharmacological interventions on cognition.

